# A golden age of behavioural social psychology? Towards a social psychology of power and intergroup relations in the digital age

**DOI:** 10.1111/bjso.12896

**Published:** 2025-04-29

**Authors:** Mark Levine

**Affiliations:** ^1^ Department of Psychology Lancaster University Lancaster UK

**Keywords:** behavioural social psychology, digital technologies, power, resistance, social identity

## Abstract

This paper explores the idea of a ‘golden age’ in social psychological research. I begin with ‘behavioural social psychology’—research that leverages the behavioural traces that are a product of the digital age. I argue that the ability to analyse digital visual data, natural language data, and smartphone and ambient sensor data, has made substantial contributions to the state of social psychological knowledge. However, social psychology needs to do more than just leverage digital data for psychological benefit. Digital technologies construct and reflect a world that is marked by profound structural inequality and unfairness. Yet social psychology never really considers technology as being ‘world‐making’ in its own right. More specifically, social psychology very rarely goes beyond considering what technology might do—to explore the question of who wins and who loses when technologies reshape our worlds. I point to a mosaic of work applying social identity approaches to new technologies as the starting point for a social psychology that engages with power and resistance in the digital age. Social psychology will not enter a truly golden age until we engage not only with the data, but also with the power structures of digital technology.

## BACKGROUND

In a recent paper in *Proceedings of the National Academy of Science* (PNAS), Buyalskaya et al. (2021) make the bold claim that we are living though a ‘golden age in social science’ research. They argue that this golden age is a product of the availability of new digital data, the development of new analytic tools, and the proliferation of interdisciplinary research teams tackling big social problems. In this paper I explore the role that social psychology (as a social science) can play in this new golden age. I focus in particular on what I will call ‘behavioural social psychology’‐ psychological research that leverages the behavioural traces that are a product of the digital age. I will show how the availability of digital data streams—including digital visual data, naturally occurring text data, and mobile and wearable sensor data—have transformed our ability to record human activity. The ability to render everyday behaviour ‘visible’—and to create a veridical record of those ordinary interactions that can then be revisited at the researcher's leisure—offers an unprecedented opportunity to advance psychological knowledge. At the same time, it presents us with a series of moral, ethical and practical challenges to address. These challenges are echoed across the social sciences, but I will focus here on the implications for social psychology—and where, as a discipline, social psychologists have contributions to make, and lessons to learn. I will argue that digital traces have allowed the study of behaviour to return centre stage in social psychology, but that we have been insufficiently curious about how these very technologies structure our social relations. More specifically, I will suggest that social psychology needs to embrace the study of digital technologies, not for what the technologies can do, but for who they do things to, and for who they do them for. This question of the power dynamics of new technologies, and how they advantage some and disadvantage others, is not a topic on which social psychology currently has much to say. A truly golden age for social psychology depends on our willingness to engage with the way digital technologies can make, and remake, our world.

## WHAT HAPPENED TO BEHAVIOUR IN SOCIAL PSYCHOLOGY?

Over the course of its history, social psychology has taken a rather eclectic approach to the concept of ‘behaviour’. As Jarrett (2008) points out, the discipline is built less on theory, and more on the rock of a series of classic experiments. In the early years, these ‘classic studies’ (Smith & Haslam, 2017) focussed on understanding and explaining behaviour in a number of politically and morally charged domains, and often required elaborate and highly choreographed cover stories in which behaviour could be observed and recorded. Early experimentalists such as the Sherifs, Zimbardo, Milgram, Darley and Latane designed their studies to deal directly with problems coming from the outside world. The behaviours in question included engaging in intergroup violence, inflicting punishment or humiliation on others, giving potentially lethal electric shocks to strangers, or failing to help others in emergencies.

However, as the discipline professionalized and concentrated on trying to influence social policy, the definition of what counted as behaviour became more abstruse (Pettigrew, 2020). Social psychology became more absorbed by technique (Sullivan, 2020) and more internally self‐referential (Smyth, 2001). The concept of behaviour stretched to include introspective reflections, questionnaire ratings, and computer keyboard presses (see also Doliński, 2018; Furr, 2009). Baumeister et al. (2007) describe this shift as leading to a ‘science of self‐reports and finger movements’. While there is clearly merit in exploring the cognitive dimensions of human social interactions, the drift away from engaging with actual behaviour (rather than its proxies) has been damaging for the relevance (and the science) of social psychology (Berkman & Wilson, 2021; Parker, 2013).

One part of the decline in focus on real‐life interactions was a recognition of the ethical challenges bound up with exposing participants to moral, physical, or psychological jeopardy. The development and enforcement of codes of research ethics began to reshape the kinds of behaviours that could be studied. For example, the first American Psychological Association (APA) Ethical Principles of Psychologists and Code of Conduct was published in 1953 and then updated on ten occasions over the next 50 years (APA, 2010). As ethics review boards began to police what was considered ethically acceptable research ever more tightly (Schrag, 2010), researchers inoculated themselves against failing ethics reviews by focusing more on self‐report or proxy simulacra, rather than the acts themselves.

## BEHAVIOURAL RESEARCH IN THE DIGITAL AGE

The advent of the digital revolution has been a game‐changer for behavioural research. We now inhabit a world in which almost everything we do leaves some kind of digital trace (Rafaeli et al., 2019). This means that the kinds of behaviours that the ‘classic studies’ were set up to collect are now automatically recorded in a variety of digital forms. The digital surveillance of public (and sometimes private) spaces through CCTV systems, body‐worn cameras, car/bike/helmet cameras, and individually directed smartphone filming, frequently captures behaviours in emergency situations that involve prejudice, aggression, violence as well as range of prosocial acts. At the same time, the proliferation of (and participation in) social media and other digital platforms means that people are also constantly producing and leaving digital traces of their everyday activities. There has been an explosion of text and visual data through social networking, social media and video sharing platforms, as well as blogging and messaging platforms. These data also capture the kinds of sensitive interactions that early social psychologists of behaviour were concerned with. Finally, this visual and textual data can also be accompanied by the availability of other sensor data—usually derived from the ubiquity of the mobile phone. Smartphones and wearable technologies track people's geographic coordinates via GPS, as well as their physical activity, such as speed of walking and rotation (via accelerometers and gyroscopes). This information about human movement patterns can be combined with data about what or whom people are interacting with on a moment by moment basis, to generate a powerful lens with which to interrogate sensitive social problems.

## SWAPPING ETHICAL CONUNDRUMS

The allure of these digital traces is that they offer unobtrusive observational data, collected without any researcher‐derived ‘production’ of behaviour. This reduces the potential for findings to be contaminated by ‘demand characteristics’. At the same time, people are also not aware that their data are being captured or have become so accustomed to this data collection that it no longer changes their behaviour. Thus, digital data is largely free of ‘reactivity’. Digital data also opens a window on behaviour in highly charged ‘emergency’ events, containing threat, peril or actual violence and is often the only way (ethically or practically) to study such phenomena directly. Taken together, digital data offers the possibility of high verisimilitude and access to behaviour in the kinds of real world, high stakes environments that the early social psychologists saw as central to the discipline.

However, the fact that this digital capture of behaviours occurs without researcher intervention does not mean that the ethical challenges evaporate. On the contrary, there has been an explosion of recent writing on the challenges facing those trying to work ethically and responsibly in the new digital data landscape. Early attempts to defend the ethics of using such data simply because ‘it is already in the public domain’ have been thoroughly discredited (Zimmer, 2010, 2018). Researchers have grappled with how the principles enshrined in the influential Belmont Report (1979) – of respect for persons, beneficence and justice – should be understood in the digital age. For example, a recent special issue in American Psychologist on *Ethical Challenges in the Use of Digital Technologies in Psychological Science* (Light et al., 2024) curates 10 papers that explore different ethical challenges in respect of individuals and groups that are socially, legally, physically or politically vulnerable. They include consideration of research that uses digital visual data (Levine et al., 2024), language technologies (Diaz‐Asper et al., 2024) and mobility data using smartphones (González et al., 2024) as part of this review. Their conclusion is that we are working in a period of ethical uncertainty—particularly in the context of digital behavioural data. While there have been a number of more recent attempts to enshrine codes of ethics that embrace digital data use e.g. by the Heise et al., 2019; the British Psychological Society, (Oates et al., 2021), the landscape is constantly evolving. This has led some researchers to develop decision making tools to help researchers navigate the ethical complexities at every stage of digital behavioural research from conceptualisation through to the final written manuscript (see Shaw et al., 2023).

## TRANSFORMING THE RESEARCH LANDSCAPE: ADVANCES IN SOCIAL PSYCHOLOGY KNOWLEDGE USING THREE TYPES OF DIGITAL DATA

At the same time as the ethical landscape is evolving, researchers are already using digital data to make significant advances in several areas. In this section, I will consider three types of digital data and their application to three core topics in social psychological research. First, I will examine the contribution of digital visual data to the study of behaviour in emergencies. Second, I will examine the insights that natural language analysis of social media data has contributed to the study of social identity. Third, I will consider location sensing technologies (contained in smartphones for example) and what they can tell us about prejudice reduction and the contact hypothesis.

### Digital visual data and behaviour in emergencies

Over the last 50 years, there has been debate about the nature of human behaviour in emergency situations. The ‘myth of panic’, which dominated discourse about behaviour in emergencies has been challenged by scholars in disaster management, sociology as well as social psychology (Drury, 2018). Researchers attempted to counter the assumption (which had made its way into emergency planning and scenario modelling) that humans would behave in an irrational, selfish and unpredictable way when faced with a life‐threatening emergency. They did so using a variety of documentary, observation or self‐report methods. The fact that this research always relied on the self‐report of participants or the judgements of individual researchers, ensured that the evidence was never strong enough to refute the ‘panic’ idea completely. However, the rise of public space CCTV surveillance and other digital visual data from body‐worn cameras to smartphones, has meant that footage from all kinds of emergency situations is now available. This data has been used to conduct robust and systematic analysis of the range of actual behaviours that are exhibited by ordinary people caught up in the emergency. This has transformed how we understand behaviour in emergencies. For example, Linderoth et al. (2015) analysed the behaviour of witnesses to cardiac arrests captured on CCTV in Copenhagen. Burroughs and Galea (2015) and van der Wal et al. (2021) analysed footage of people seeking to escape fires in a variety of settings. Lambie et al. (2016) analysed footage of human behaviour during and immediately following earthquake shaking. Philpot and Levine (2022) studied evacuation behaviour in a subway train emergency. Au‐Yeung et al. (2024) analysed footage of the spontaneous public response to a marauding knife attack on the London underground. Bernardini and Quagliarini (2021) analysed footage from terrorist attacks across Europe. This growing body of digital video data—which captures behaviour in emergencies first hand—world—has helped not only to bury the myth of panic, but also to provide a veridical platform for understanding the prevalence and range of actual behaviours in emergencies. A recent systematic review of the behaviour in emergencies (Philpot et al., 2025) shows conclusively that helping others, seeking shelter, seeking information and following/moving together with others are the most common behaviours in emergencies. Antisocial behaviour and expressions of fear or panic are extremely rare by comparison. This work has transformed the way emergency contingency planning and modelling is now done (Carter et al., 2020; Templeton & Neville, 2020). More specifically, it has cemented recognition of the key role of ordinary people as ‘zero responders’ or the first line of defence in any emergency response (Cocking, 2013; Drury et al., 2019).

Public space CCTV and other digital video footage (including body worn and smart phone filming) has also changed how we understand the psychology of bystanders in public spaces. The traditional view of bystander behaviour in emergencies was dominated by the idea of the ‘bystander effect’ (Darley & Latané 1968, Latané & Darle 1970). This referred to a phenomenon whereby individuals are less likely to offer help to a victim when other people are present, because of diffusion of responsibility among the group. The key idea was that the larger the number of bystanders, the lower the likelihood that any one person will intervene. Latane and Darley's work also established the idea that bystander helping levels were low and that this was an important societal problem. There have been challenges to this idea over the years, particularly from the meta‐analysis conducted by Fischer and colleagues (Fischer et al., 2011). They demonstrated that, while there was a small to medium sized bystander effect in more mundane helping situations, the bystander effect did not hold for violent or dangerous emergencies. In such situations, individuals are more likely to receive help when more bystanders are present. Fischer et al. (2011) propose a ‘reverse bystander effect’, where a greater number of bystanders increases the likelihood of intervention, particularly when the emergency is unambiguous, and it is clear what actions bystanders should take.

Analysis of incidents of public space aggression and violence captured on camera has allowed the bystander behaviour picture to become much clearer. Philpot et al. (2020) using CCTV data from Britain, the Netherlands and South Africa, show that bystander intervention rates in incidents of public aggression are high (at just over 90%). Levine et al. (2011) shows that group processes are not inimical to successful bystander intervention. Successful de‐escalation of aggression by bystanders is a function of increased group size and the collective coordination of the bystanders. Liebst et al. (2019) show that social relations and the presence of others predict bystander intervention. Ejbye‐Ernst et al. (2022) analyse bystander intervention strategies as they unfold, and reveal the kinds of bystander strategies that are more likely to be successful (Ejbye‐Ernst, 2023). Lindegaard et al. (2022) show that the likelihood of bystander intervention is not related to the potential danger level of the emergency incident and Liebst et al. (2021) show that the risk of being victimized for bystander intervention behaviour is low. Taken together, this body of empirical work on real‐life bystander behaviour provides a platform for redesigning how bystander training is conceptualized and delivered. The evidence from digital visual data demonstrates conclusively that, in more serious or consequential emergencies, the key problem is not encouraging bystanders to intervene. On the contrary, bystander intervention levels are high. What is more important is the question of how to make that intervention successful – and the key to successful intervention is the presence and coordination of the group (Levine et al., 2020).

### Natural language and Behavioural markers of social identity salience

In addition to digital visual data, there has been an explosion of natural language data from social media and other digital communications. The ubiquity of this kind of language data, combined with new methodological and computational innovations, has meant that Natural Language Processing (NLP) has grown in popularity across the social and computational sciences (Boyd & Markowitz, 2025). In psychology, the primary focus of this approach has been to use computational tools and natural language to reveal (or perhaps clarify) a range of underlying psychological constructs (including emotions, motivations, traits, personality, attitudes, status (see Tausczik & Pennebaker, 2010; Boyd & Markowitz, 2025)). Researchers engage in computational analysis of word use, with the intention of exploring an underlying psychological state. A major strength of this approach is that the analytic tools leverage the stylistic features of the language (as opposed to simply analysing the content), thus revealing the underlying structure of the text, rather than just its semantic form.

While there is work in this tradition that recognizes the impact of context on language production (see Boyd & Schwartz, 2021) it is usually assumed that individuals write in relatively stable and invariable ways based on their underlying personality, or the demographically defined groups to which they belong (e.g. genders (Newman et al., 2008), ages (Löckenhoff et al., 2008) politics (Sylwester & Purver, 2015) personalities (Mairesse et al., 2007; Pennebaker et al., 2005)). The default assumption is that there will be ‘stability’ at the level of the individual. However, there is also a tradition of natural language research which goes beyond the idea of stability, to treat language as a behavioural act in its own terms. Rather than assuming that the language reveals some underlying construct, the approach treats language in the spirit of Uher's (2016) metatheoretical definition of language as a form of behaviour—both in the ‘here and now’ and in the very act of transmission itself. By focussing on what language is doing, rather than what it is revealing, this work brings new insights about how language is related to psychological processes. For example, Koschate et al. (2021) draw on social identity theory to argue that people have multiple psychological group memberships from which they can draw social identity resources.

This social identity approach to linguistic style appreciates that individuals may identify with many different social groups which influence behaviour only when those identities are salient (Turner et al., 1987). The social identity approach suggests identity salience is a key feature that mediates these style shifts. A change in audience or topic can influence which social identity is salient, consequently impacting an individual's communication style. It thus becomes possible to explore changes in the linguistic style of the same individual as a potential behavioural marker for changes in the salience of different social identities. For example, Koschate et al. (2021), use the prototypical linguistic style of group members to detect the salience of either a parent or feminist identity in the same individual. Using different social media forums (on Mumsnet) as a proxy for identity salience, they demonstrate that it is possible to predict which forum a post from the same individual originated in, based only on linguistic style features. Further, they cross‐validate this methodology in an offline experiment where identity salience was manipulated explicitly, finding that their classifier (trained online) was correctly able to predict which identity (parent or feminist) was made salient at the point of writing. Cork et al. (2020) take a similar approach to natural language data from Reddit forums and from the darknet Silk Road. They train a linguistic measure of prototypicality for two groups: libertarians and entrepreneurs, showing that it is possible to detect posts from the same individual across forums. This work on linguistic shifts as a function of changes in social identity salience, has been combined with experimental work on the cognitive costs of social identity salience shifting (See Zinn et al., 2022, 2023). Taken together, this combination of natural language processing and experimental design provides robust evidence for one of the key (but hitherto weakly evidenced) cognitive mechanisms of the social identity approach. We can now use natural language analysis of language data to explore (potential) moment by moment changes in social identification in the same individual (and not just changes over the long duree (see for e.g. Smith et al., 2020)).

### The contact hypothesis and the use of location sensing data

In a recent paper, Hinds et al. (2022) document the many ways in which digital data about human movement is now also being leveraged for psychological study. They review the kinds of digital data and sensor technology that have been used to track human mobility. This includes GPS tracking with geographic information system (GIS) analytics, mobile phone location, Bluetooth encounters between devices, geotagging of tweets, and SMS origins and destinations. They catalogue the way digital data about location, proximity, finances, accelerometry, orientation, and social network can be used to unpick movement patterns. They show that researchers have often combined these digital movement recording techniques with more traditional experience sampling methods to gain greater insight into the relationship between movement patterns and psychological experience.

Where the mobility tracking technology has proved most transformative in social psychology is in the study of intergroup contact and prejudice reduction. Research has long extolled the benefits of intergroup contact as a means of reducing prejudice and promoting positive intergroup relations (Pettigrew & Tropp, 2013). However, McKeown and Dixon (2017, p. 10) have cautioned against relying on evidence that is derived from research in controlled settings and standardized self‐report or survey methods as they may miss important information about ‘the nature, meaning, and consequences of contact *on the ground*’. For example, Dixon et al. (2020) point to a growing body of research suggests that formal policies of desegregation are often offset by informal ‘micro‐ecological’ practices of (re)‐segregation, in everyday life spaces. They show, using digital tracking technology, that even though a formal agreement to end the ethno‐nationalist conflict in Northern Ireland (the ‘Good Friday Agreement’) came into effect more than 20 years ago, the psychological divide between Catholic and Protestant spaces in Belfast still exists. They tracked the everyday movements over time of 233 Catholic and Protestant residents of north Belfast over a two‐week interval. Using a custom mobile phone app to gather space time point estimates at a rate of one data point per 4 seconds, their approach yielded almost 1000 h of mobility data, based on over 22 million GPS points, and captured residents' journeys to over 5000 destinations. Participants augmented this data through taking photographs (more than 1000 images) and engaging in ‘walking interviews’. By analysing the movements patterns, and integrating coding of the images and interview data, they were able to reveal the sectarian constraints placed on Protestant and Catholics day‐to‐day movements in Belfast. These constraints have continued to divide communities some 20 years after the Good Friday peace agreement and to hamper residents' ability both to move freely through the city and benefit fully from its public facilities.

In similar fashion Keil et al. (2020) designed a bespoke app to act as a contact logger for everyday contact in public space. Their location aware mobile application also had the capacity to record the quality (both positive and negative) of every contact experience. They used the ‘Contact Logger’ to explore contact between generations, in particular older and younger people. Their usability study showed that it was possible to develop pictures of the interactions between location and the quality of outgroup contact (from positive to negative). This more dynamic picture of the ‘locatedness’ of contact helps us to understand how geographical and architectural variables moderate the effect of contact on intergroup relations. It can also aid the design effective interventions embedded in individuals' everyday experiences, for instance through the optimization of public interaction spaces.

## GOING BEYOND DIGITAL DATA FOR PSYCHOLOGICAL BENEFIT: WHY STUDYING THE EFFECTS OF TECHNOLOGY IS NOT ENOUGH

Thus far we have seen how engaging with digital data can advance psychological knowledge. The focus has been on examining how we can use computers and digital data to extract psychologically relevant information. We have seen different ways in which this digital behavioural data can be related to grander theories in social psychology. We have also explored some of the ethical challenges that surround the use of digital data in psychological research. However, the interrelationship of technology and psychology goes much deeper than just the leveraging of digital data for psychological benefit. Social psychologists already study the effects of technology on individual behaviour. For the most part, this approach to research is usually framed by negative affect: i.e. psychologists begin with concerns about the dangers of new technologies. For example, there is a large literature on the effects of technology on health and well‐being. Of particular concern is whether digital technologies and social media affordances produce negative health behaviours including addictions (Sun & Zhang, 2021), self‐harm (Dyson et al., 2016) and even suicide (Sedgwick et al., 2019; Twenge et al., 2018). Researchers also explore the effects of technologies on civic health and public order. This work examines issues like increasing social and political polarization (Van Bavel et al., 2021), the spread of conspiracy theories (Douglas et al., 2017) and the problem of radicalisation (Mølmen & Ravndal, 2021). While the general tenor of this research is concerned with the potential harms that technology might be facilitating, there are scholars who challenge the threat narrative. For example, Etchells (2024) and Ellis (2019) write extensively on the questionable provenance of ‘technology addiction’; Gill (2015) argues that radicalisation is not simply an internet‐facilitated phenomenon, and Uscinski et al. (2018) argue that the rise and spread of conspiracy theories is not a simple product of the development of digital technologies. Finally, there is a research current that takes a more practical focus, being concerned solely with the implementation of new technologies. This work seeks to use psychological insights to facilitate technology adoption. The tenor of this work is that new technologies are to be welcomed, but that people are often reluctant to accept them. This might be because there are particular beliefs (Agarwal & Prasad, 1999), emotions (Di Giacomo et al., 2019; Gerli et al., 2022) or other psychological barriers to adoption (Adams et al., 2005). Researchers also explore barriers around perceptions of trust in technology (Choung et al., 2023), the threat technologies might play to traditional ways of operating (Carpenter et al., 2019) and the relative risks people imagine as a consequence of engaging with new technologies (Horst et al., 2007).

## TECHNOLOGY, POWER AND INTERGROUP RELATIONS: WHY SOCIAL PSYCHOLOGY SHOULD BE MORE ‘LUDDITE’

This focus on the negative or positive effects of technology tends to obscure the power dynamics and intergroup relations in which the technologies are embedded. As Doctorow (2024) argues, when we focus on the affordances of technology alone, we neglect one of the most important questions of the digital age. He writes ‘technology isn't the problem…… we should stop thinking about what technology does and start thinking about who technology does it to and who it does it for’. Social psychology needs to move beyond the ‘sisyphean cycle of technology panics’ (Orben, 2020) to consider how new technologies exploit or transform existing social relations in the worlds we inhabit. Take as an example the history of a previous technological revolution—the story of the ‘Luddites’ and the invention of the mechanized power loom during the British industrial revolution. Power loom mechanization began in the late 18th Century and radically transformed textile production—changing both working practices and social relations. In the early 19th Century, a secret society styling themselves ‘the Luddites’ began to smash textile machinery in the mills of England. Luddites were portrayed as opponents of technological progress, rejecting a new technology because it replaced skilled weavers with unskilled machine operators. Today, we use ‘luddite’ as a pejorative referring to people who are backwards, anti‐technology reactionaries. However, as Thompson (1963) argues, the Luddites' rejection was not driven by technophobia, but rather an understanding of whose interests the new technologies were serving, and who was being exploited as a result. They argued that the new machines could have allowed workers to produce much more cloth in far fewer hours, at a lower cost, all while being paid fairly. The reduced cost per unit could have been balanced out by higher sales volumes, and increased production could have been achieved in fewer working hours. All of this could have benefitted the lives of workers. Instead, factory owners—whose wealth had been built on the hard work of textile labourers—chose a different path. They hired fewer workers (meaning increased poverty and starvation), kept the same long hours, paid lower wages to retained workers, and kept the significant savings for themselves. In wrecking the machines, the Luddites were not against technology per se. They wanted a more equitable distribution of the benefits of the new technology. Smashing looms and stocking frames was the Luddites' *tactic*, not their goal. The Luddite movement was an example of collective action in response to injustice, and the behaviour exhibited was something Hobsbawm (1952) calls ‘collective bargaining by riot’. Social psychology can learn from this Luddite sensibility. The Luddites were not reflexively anti‐technology. They were skilled artisans who had a history of incorporating new technologies into their profession (Merchant, 2023). They argued for, and then fought for, a future in which the benefits of technology were distributed fairly. They lost that battle, and history has maligned them. However, the lesson that Luddism bequeaths to social psychology is the importance of focussing on the social consequences of the way technologies are deployed. Social psychology needs to engage more with how technologies shape the distribution of health, wealth and opportunity in both the digital present and digital future.

## WHERE IS THE SOCIAL PSYCHOLOGY OF TECHNOLOGICAL POWER AND RESISTANCE?

This question of who new digital technologies advantage, and who they disadvantage, is not something that social psychologists have paid much attention to. Outside social psychology, fierce debate ranges about the winners and losers of the digital age. For example, in the sphere of politics and economics, this has catalysed around the publication of Zuboff's (2019) *The Age of Surveillance Capitalism*. Zuboff argues that the digitisation of everything gives technology firms immense social power, and that we have entered a new era of ‘surveillance capitalism’—where human emotion and experience is expropriated as ‘behavioural surplus’ to fuel the capitalist machine. Her critics argue that her theorisation of capitalism is weak (Morozov, 2019) or that her understanding of surveillance is less than novel (Ball, 2019), but her work still establishes the importance of understanding the way digital technologies in general, and algorithmic surveillance in particular, are impacting the lives of modern humans. Scholars including Noble (2018), Eubanks (2018), O'Neil (2017) and Benjamin (2019) have already mapped some of the negative implications of algorithmic surveillance and algorithmic bias for Black, working class, female and other minority or disadvantaged groups.

This apparent lack of concern among social psychologists about the power dynamics of digital technologies is particularly surprising given the emphasis social psychologists put on the impact of social context on psychological behaviour. For example, Reicher (2004) argues that the social identity tradition ‘is based on an insistence that human action needs to be understood in its social context… the social identity tradition forces us to turn towards the social world. It forces us to address the ideological and structural features of that world’ (p 921). In studying prejudice, discrimination, disadvantage and other forms of social conflict, social psychologists see the impact of social context in the presence of other groups (Drury & Reicher, 2000), the physical location of the action (Dixon & Durrheim, 2000) the influence of history (Liu & Hilton, 2005), politics (Huddy, 2001), time (Cinnirella, 1998), culture (Hopkins & Reicher, 2011), economics (Jetten et al., 2017) and ideology. (Devine, 2015). What is entirely absent from this list of social contexts is technology. Despite the ubiquity of digital technology, and the deep penetration into all corners of human life, social psychology never really considers technology as being ‘world‐making’ in the way that it imagines these other contextual forces to be. More specifically, it very rarely goes beyond considering what technology might do—to explore the question of who it does it to, and who it does it for.

## SOCIAL IDENTITY, TECHNOLOGY, POWER AND RESISTANCE

From a psychology perspective, the workings of power in a social context are often revealed by acts of resistance (Haslam & Reicher, 2012; Reicher, 2004; Reicher & Levine, 1994). Outside psychology, researchers are already exploring the ways in which ordinary people engage in resistance to the power of digital platforms and their algorithms. For example, Bonini and Treré (2024) explore the ways in which gig and platform workers (e.g. delivery couriers or Uber drivers), people in the cultural industries (e.g. artists and content creators) and political and social activists, navigate the ideological and structural features of the technological world in which they exist. They describe how gig workers collaborate in an attempt to ‘game’ the algorithms that seek to govern them. For example, they discuss solidarity log‐outs by courier drivers in Mexico and China, co‐ordinated order refusals by US Doordash drivers, and more traditional forms of worker street protest organized through encrypted messaging services outside the platform. When it comes to resistance in the cultural industries, they describe collective action to ‘game’ platforms like Instagram through the creation of ‘engagement pods’—grassroots communities that agree to mutually like, comment on, share, or other‐ wise engage with each other's posts, no matter the content. This uses the affordances of the algorithm to benefit of the producers—rather than the platform itself. Finally, they describe the battleground of ‘algorithmic activism’ where forces across the political spectrum (including state actors, political groups, and more informal ‘opinion based’ groups) are in an ever‐changing battle with the platform to have their content promoted, to make a message go ‘viral’, or to deny the platform to others. What this body of work recognizes is that the power of technology is not unidirectional. As Bonini and Trere argue, ‘users and platforms, human and nonhuman subjects co‐inhabit this ecosystem in complex ways…. Alliances with algorithms and rebellions against or through them alternate incessantly in everyday life’ (2024, p. 160).

## SOCIAL IDENTITY AND SOCIO‐TECHNICAL SOCIAL PSYCHOLOGY

While there is, as yet, very little social psychological work that engages with these everyday acts of technology‐orientated resistance, there is the beginnings of a social identity toolkit that may be of use. For example Stuart and Levine (2017) and Stuart et al. (2019) have written on social identity, technology and privacy; Bingley et al. (2021) offer a social identity theory of information—access regulation (SITAR) with implications for digital technologies; Cooper (2020) has written on social identity and algorithmic surveillance; Mirbabaie et al. (2021) have written on social identity and collaboration with virtual assistants; Kordoni et al. (2023) and Gavidia‐Calderon et al. (2023, 2024) write about social identity and semi‐autonomous agents like drones and robots; and Bingley et al. (2023) explore social identity and the development of human‐centred AI. This mosaic of work applying a social identity approach to the impacts of new technologies is one starting point for the development of a social psychology that is actively engaged with the world we inhabit. The world‐making properties of technologies including AI, autonomous agents, nanotechnologies and quantum computing need a social psychological lens that is engaged not simply with the potential effects of technologies, but with the power relations that are embedded in their proliferation. If our discipline continues to see these kinds of questions as being the province of others in the social sciences, then we risk irrelevance. To contribute fully to the ‘golden age of social science research’, we need to build a socio‐technical social psychology.

## SOCIAL PSYCHOLOGY AND INTERDISCIPLINARY RESEARCH TEAMS

In their paper on the ‘golden age’ of social science research, Buyalskaya et al. (2021) make strong claims for the rise of interdisciplinary teams to tackle big social problems. How have social psychologists faired in this new landscape? Are social psychologists open to, and engaged in, interdisciplinary teams? We have already seen some of the successes in the sphere of privacy (Bingley et al., 2021; Stuart et al., 2019; Stuart & Levine, 2017), humans and autonomous agents (Gavidia‐Calderon et al., 2023, 2024; Kordoni et al., 2023), and human‐centred AI (Bingley et al., 2023). However, there is much more to do in the realm of power relations and social justice. While social psychologists write extensively about big social problems including prejudice, discrimination, bias and social inequality, they are very rarely part of interdisciplinary teams exploring these topics in the contexts of digital technologies. Scholars in data science disciplines now clearly recognize the way algorithmic technologies constantly produce (or reproduce) unfair or unequal outcomes, including blatantly racist, sexist, classist, ageist and ableist algorithmic outputs (Broussard, 2018; Noble, 2018). For the most part, data scientists tend to see this as a problem of ‘bias’ in data sets, something that can be dealt with by trying to tweak, or correct the data set. But this language of bias doesn't help us to understand how to deal with the pre‐existing social inequalities that are reflected in the data. We can't fix society in the same way we might ‘de‐bias’ a data set. As Zajko (2022) argues, ‘when the truth is that society is deeply, structurally unjust and unequal, and that technologies are part of these structures, the question is whether our algorithms should accurately reproduce inequality or work to change it.’ (p4). It is here that social psychological expertise has the potential to be most valuable. There are long established traditions in social psychology on how to challenge bias, and to create the conditions for social change that can lead to fair and equitable outcomes. If social psychologists were more willing and able to engage their conceptual toolkit as part of interdisciplinary teams engaged in the study of digital technologies, they would be in a position to make important contributions. They could add value to the growing body of AI researchers who are attempting to shift data science away from just trying to make data bias and inequality transparent, to a more radical path of trying to tackle the underlying relations of power (see for example Bender and Friedman (2018), Buolamwini and Gebru (2018), Mitchell et al. (2019), Gebru (2020), Birhane (2021), Birhane et al. (2022)).

## CONCLUSION

I began this paper with a question about a potential golden age of social science research, and the place of social psychology within it. I hope I have demonstrated that there are several ways in which social psychology is making an important contribution to this idea of a golden age—particularly in respect of the new ethics required for research with digital data. I also offer evidence that the analysis of digital visual data, natural language processing, and smartphone and ambient sensor technology data has made substantial contributions to the state of social psychological knowledge. It is also clear that social psychologists are now embracing computational methods and techniques to analyse complex data sets, and contributing a social psychological toolkit to work in collaboration with colleagues from linguistics, sociology, law, political science, computer science and software engineering. This combination of digital data, new computational techniques, and interdisciplinary team working fits well with Buyalskaya et al. (2021) conception of a golden age.

However, I also argued that there is an important lacuna at the heart of social psychology in respect of this golden age. While we might be using digital data as a way to reveal psychological concepts, or study the effects of digital technology on individual behaviour, we almost never explore the power dynamics of technological proliferation. Put another way, the discipline of social psychology, which places the study of ‘context’ at the core of its mission, is yet to recognize that ‘technology’ might be a context in itself. In making this case I am aware that there is a body of work on the similarities and differences between behaviours exhibited online vs. offline (e.g. Lieberman & Schroeder, 2020) or on behaviours in virtual or augmented reality (e.g. Blascovich et al., 2002; Fox et al., 2009). My point is that these are studies of what technology does – not who it does it too, and who it does it for. I am arguing for a much more fundamental approach to the importance of technology from a social psychological perspective. Digital technologies are (as technologies always have been) ‘world making’ phenomena. They construct the world in which we live, and that world is marked by profound structural equality and unfairness.

We are living in an era that some have described as the ‘Fourth Industrial Revolution’ (Philbeck & Davis, 2018). Technologies including artificial intelligence, quantum computing, gene editing, nanotechnologies, autonomous agents and advanced robotics, will all continue to blur the lines between physical, digital and biological worlds. Like all revolutions, these technological developments will afford social, political and economic change, as well as psychological transformations. And as with all revolutions, there will be struggles over the values that should shape the worlds which emerge. Social psychology in general, and social identity theorists in particular, with their long history of work on intergroup relations, prejudice, discrimination, power and resistance, are well placed to make contributions to the interdisciplinary research teams tackling these big societal questions. However, if our discipline continues to see these kinds of questions as being the province of others in the social sciences, then we risk irrelevance. To contribute fully to the ‘golden age of social science research’, we need to build a socio‐technical social psychology that is up to the challenge.

## AUTHOR CONTRIBUTIONS

**Mark Levine:** Conceptualization; funding acquisition; writing – original draft; writing – review and editing.

## CONFLICT OF INTEREST STATEMENT

I have no conflicts of interest to declare.

## Data Availability

Data sharing not applicable to this article as no datasets were generated or analysed during the current study.
